# Microcirculatory Disorders and Protective Role of Xuebijing in Severe Heat Stroke

**DOI:** 10.1038/s41598-018-22812-w

**Published:** 2018-03-14

**Authors:** Hui Jin, Yi Chen, Chengjia Ding, Youping Lin, Yulan Chen, Dongxin Jiang, Lei Su

**Affiliations:** 1 Department of ICU, General Hospital of Guangzhou Military Command, Key Laboratory of Tropical Zone Trauma Care and Tissue Repair of PLA, Guangzhou, 510010 China; 2 Department of Critical Care Medicine, The Fifth People’s Hospital of Dongguan, Dongguan Hospital Affiliated with the Medical College of Jinan University, Dongguan, 523900 China; 3 Shantou University, Shantou, 515000 China; 4 The Fifth Affiliated Hospital of Guangzhou Medical University, Guangzhou, 510000 China

## Abstract

This study was conducted to explore underlying mechanism of microcirculation dysfunction and protectiverole of Xuebijing in heat stroke. Forty rats were divided into: control, vehicle + heat stress (HS), superoxide dismutase (SOD) + HS, and Xuebijing + HS groups. Rats in heat stress groups were subjected to continuous heat stress in infant incubator 1 h after tail vein injection of the tested compound and spinotrapezius preparation. Velocity of blood flow through micro-vessels and vascular diameter were detected in real time. Another 27 rats were divided into: vehicle, SOD, and Xuebijing groups, then further divided into three subgroups each: control, Tcore = 38 °C, Tcore = 41 °C. Rats were sacrificed, and spinotrapezius single-cell suspensions were prepared for detecting SOD and reactive oxygen species (ROS). The results showed that heat stress decreased SOD activity, increased ROS levels, and reduced the blood flow rate. Xuebijing increased SOD activity, decreased ROS levels and exhibited a protective effect in terms of blood flow rate but was less protective than SOD. The survival time in Xuebijing + HS group was longer than that in vehicle group but shorter than that in SOD + HS group. The results suggested Xuebijing could decrease ROS levels and have protective effects in severe heat stroke.

## Introduction

Despite several decades of studies, heat stroke remains a major clinical problem with high morbidity and mortality and a high incidence of multiple organ dysfunction syndromes (MODS). Classic heat stroke primarily occurs in individuals with immune dysfunction during heat waves each year^1–4^. The average mortality rate of patients with heat stroke is 10–15%^5^ and can rise to more than 40% in severe cases with MODS^6^. Therefore, it is important to investigate the pathogeneses of heat stroke and develop effective preventive and treatment methods accordingly.

The microcirculation mainly ensures that the oxygen supply to tissues is adequate^7^. Microcirculation disorders usually present as decreased microvascular blood flow, damage to endothelial cells and high vascular permeability, lower microvascular reactivity, and leukocyte adhesion and migration. Studies have suggested that microcirculatory disorders are closely correlated with MODS and directly affect the prognosis of hemorrhagic or septic shock^8,9^. Oxygen free radicals produced in the interaction between the endothelium and leukocytes contributed to microcirculatory disorders in an ischemia–reperfusion model, similar to hemorrhagic shock^10^. A recent study also found that both pretreatment and post-treatment with anti-oxidants could attenuate microcirculation disorders^11,12^.

In severe heat stroke, microcirculation disorders are inextricably associated with pathophysiological processes including circulatory failure, tissue edema, and bacterial translocation. As reported in a previous study^13^, microcirculation disturbance occurs not only in the early stage but also before systemic hemodynamic disorders and manifests as reduced microvessel blood flow rate and increased water content and Evans blue concentration in lung tissue. Intervention with anti-oxidative agents [superoxide dismutase (SOD)]may have certain protective effects in severe heat stroke.

Xuebijing injection is a traditional Chinese medicine approved by the China Food and Drug Administration for treating systemic inflammatory diseases, such as sepsis, acute lung injury (ALI), and acute kidney injury. Xuebijing consists of extracts from five Chinese herbs: Carthami Flos, Paeoniae Radix Rubra, Chuanxiong Rhizoma, Salviaemiltiorrhizae, and Angelicae Sinensis Radix. A recent report identified that some active ingredients in Xuebijing, including enkyunolide I, safflor yellow A, oxypaeoniflorin, and benzoylpaeoniflorin, inhibit the activity of nuclear factor kappa B, a key factor in inflammatory responses. It was previously reported that Xuebijing inhibited inflammatory responses, attenuated liver injury, and improved survival rates in rats with heat stroke^14^. Xuebijing is likely a potential effective medicine in treating heatstroke, but studies that address the roles of Xuebijing in heat stroke are insufficient. Additionally, the roles of Xuebijing in microcirculatory disorders during heatstroke require further exploration. We hypothesize that Xuebijing is a protective agent that can attenuate microcirculation disorders during heat stroke by reducing ROS.

Therefore, in this study, we reproduced the classic severe heat stroke rat modeland evaluated SOD and ROS levels during heat stress (HS), observed the changes in the microcirculation during heat stress, and investigated the role of oxidative stress in heat stroke by comparing the effects of SOD and Xuebijing during HS.

## Results

### Changes of SOD activity in spinotrapezius tissue homogenates during heat stress

**Figure 1:**
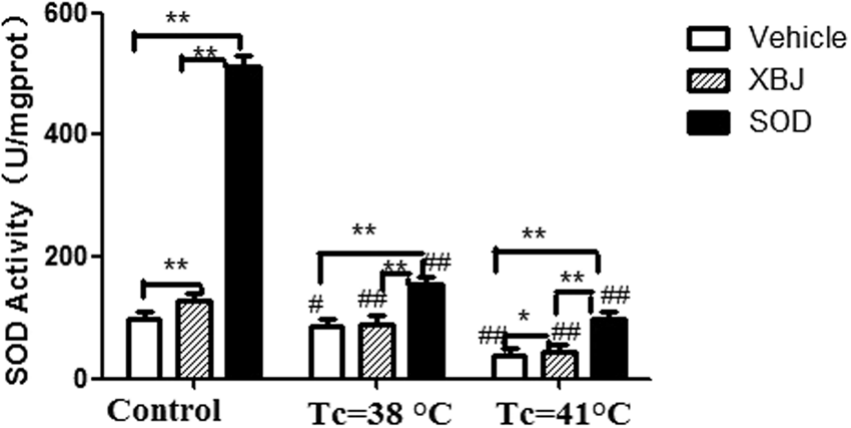
Changes of SOD activity in spinotrapezius during heat stress (HS). The SOD activity in each group was measured using corresponding kits. *P < 0.05, **P < 0.01, comparison among different treatment groups at the same heat stress temperature; ^#^*P* < 0.05, ^##^*P* < 0.01, comparison among different temperature subgroups within the same treatment group.

SOD activity decreased gradually with the increase in the core temperature during heat stress compared with the control group. The SOD activity in the Xuebijing group was significantly higher than that in the vehicle group but lower than that in the SOD group (Fig. 1).

### Changes of ROS levels in spinotrapezius single-cell suspensions during heat stress

The ROS levels in the spinotrapezius of rats in the vehicle and Xuebijing groups increased significantly with the increase in the core temperature compared with the control group (*P* < 0.01). However, the increase in the ROS level in the spinotrapezius was not statistically significant until the core temperature reached 41 °C in the SOD group (*P* < 0.01).

**Figure 2:**
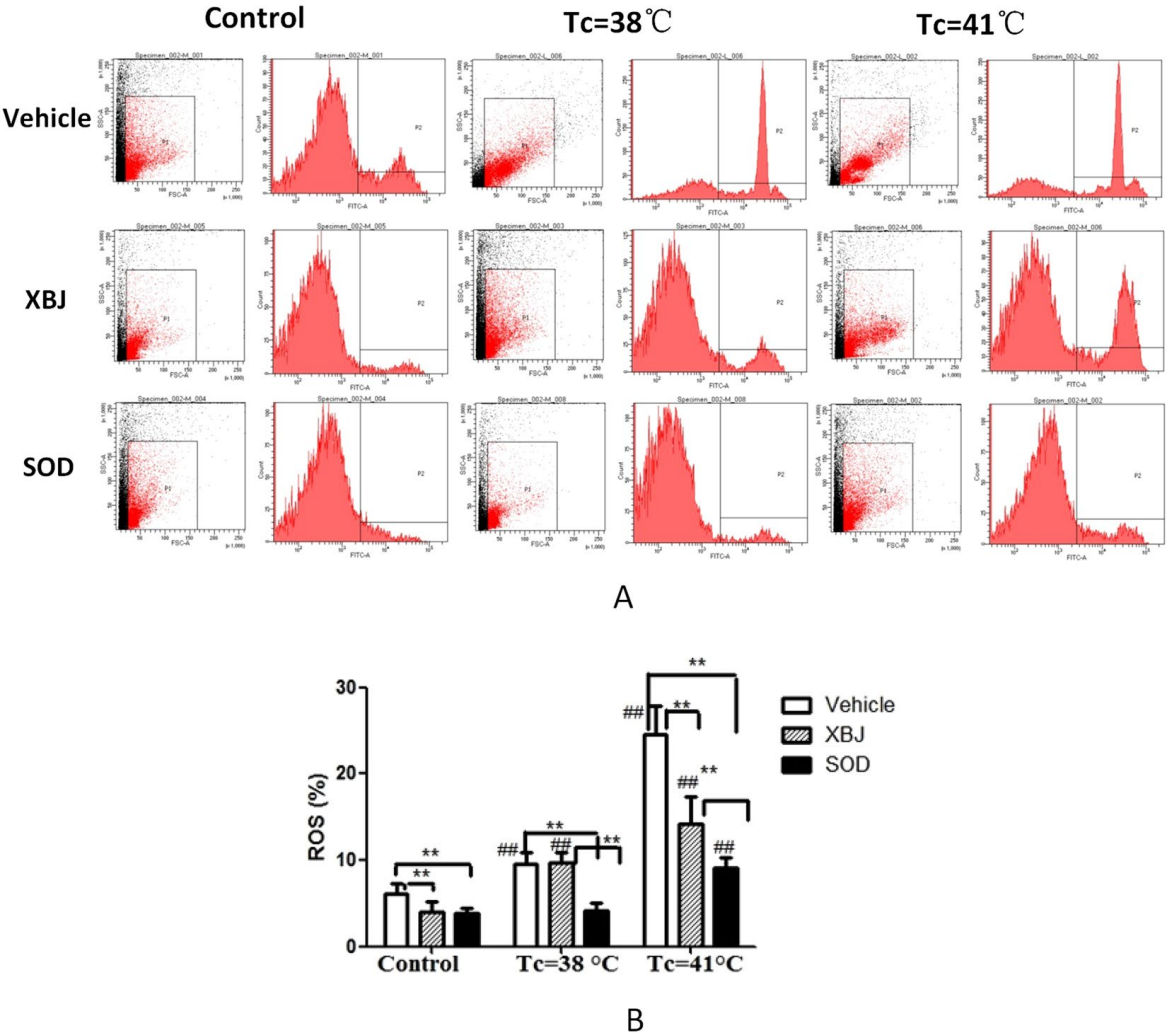
Detection of ROS in spinotrapezius by flow cytometry. (**A**) The cell numbers were high, the density distribution was normal, and the results were reliable. (**B**) Comparison of ROS levels between different groups, *P < 0.05; **P < 0.01 comparison among different treatment groups with the same core temperature. ^#^*P* < 0.05; ^##^*P* < 0.01 comparison among groups with different core temperatures but the same treatment.

The ROS level in the spinotrapezius was significantly higher in the vehicle group than in the Xuebijing and SOD groups (*P* < 0.01) (Fig. 2).

### Altered microcirculation

**Figure 3:**
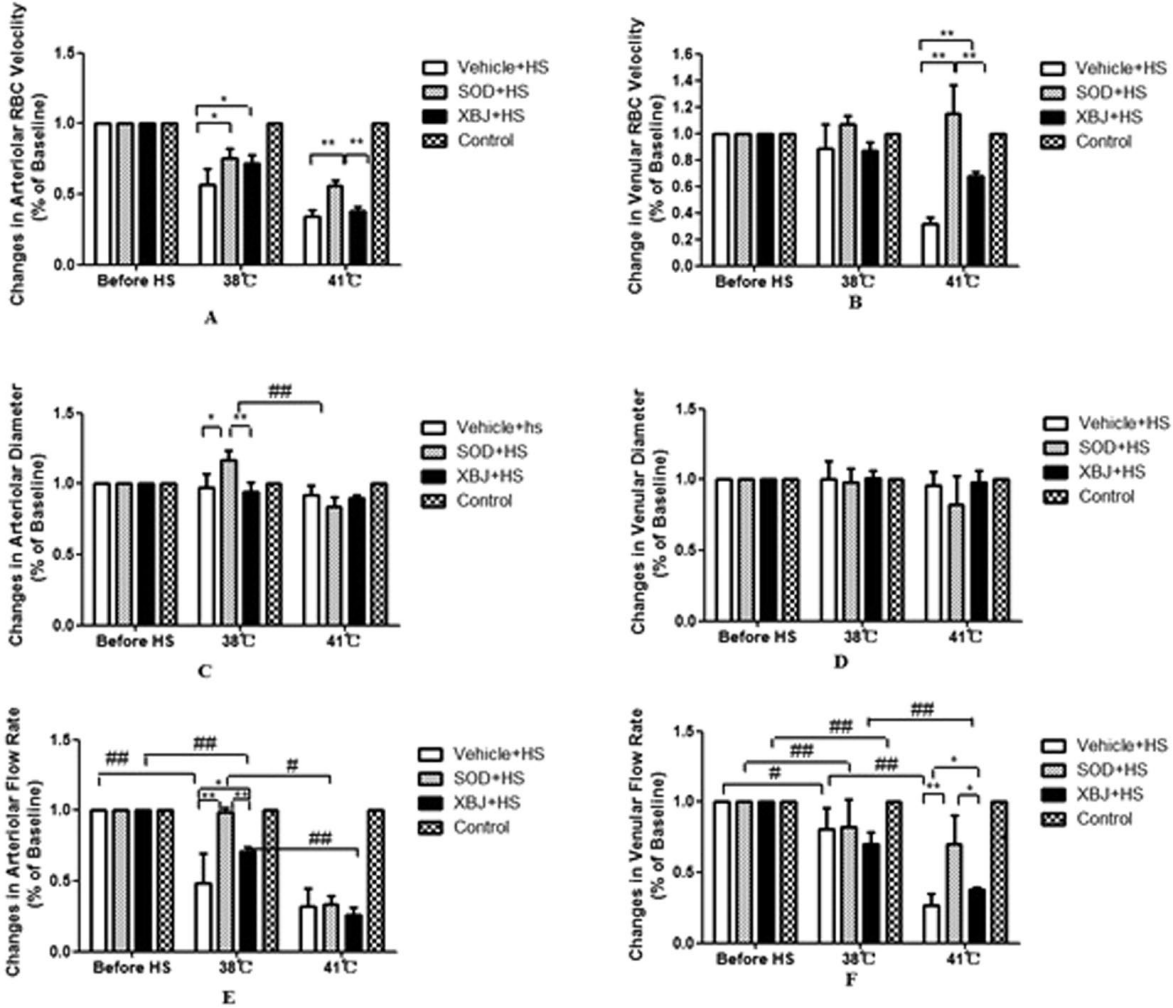
Alterations in microcirculatory blood flow. The arteriolar and venular red blood cell velocities (V_RBC_) (panels A and B) and vessel diameters (panels C and D) were assessed at a rate of 25 frames/s using a CR5000X2 high-speed camera. Rats in control group didn’t be subjected to continuous heat stress in an infant incubator, but were observed at same time of heat stress group rats. The arteriolar and venular blood flow rates (panels E and Panel F) were calculated using the following formula: π × Vmean × (D/2)^2^ when V_mean_ = V_RBC_/1.6. *P < 0.05; **P < 0.01, comparison among different treatment groups at the same core temperature. ^#^*P* < 0.05; ^##^*P* < 0.01, comparison between groups with different core temperatures but the same treatment.

Arteriolar V_RBC_: The V_RBC_ in each group decreased significantly with core temperature after heat stress compared with the control group (*P* < 0.05). The V_RBC_ decrease in the three groups of rats that exposed to heat stress was evident even when the core temperature reached 41 °C (*P* < 0.01). The V_RBC_ in the Xuebijing + HS and SOD + HS groups was significantly higher than that in the vehicle + HS group when the core temperature was 38 °C (*P* < 0.05). In addition, the V_RBC_ in the SOD + HS group was also significantly higher than that in the vehicle + HS and Xuebijing + HS groups when the core temperature was 41 °C(*P* < 0.01) (Fig. 3A).

Venular V_RBC_: The venular V_RBC_ was not significantly different between the rats that received SOD and rats in the other groups before heat stress. During heat stress, the venular V_RBC_ in the vehicle and Xuebijing + HS groups decreased significantly with the increase in the core temperature compared with the control group (*P* < 0.01). When the core temperature of rats reached 41 °C, the venular V_RBC_ in the Xuebijing + HS and SOD + HS groups was significantly higher than that in the vehicle + HS group (*P* < 0.01) (Fig. 3B).

Changes in arteriolar diameter: The changes in arteriolar diameter that occurred with the increase in the core temperature in the vehicle + HS and Xuebijing + HS groups were not significantly different compared with the control group during heat stress. However, the arteriolar diameter in the SOD + HS group decreased significantly when the core temperature was 41 °C (*P* < 0.01). The arteriolar diameter in the SOD + HS group was significantly higher than that in the vehicle + HS and Xuebijing + HS groups when the core temperature was 38 °C (*P* < 0.05). However, when the core temperature increased to 41 °C, the arteriolar diameter in the rats exposed to heat stress was not significantly different among the treatment groups (Fig. 3C).

Changes of venular diameter: The changes of venular diameter that occurred with the increase in the core temperature in the three groups were not significantly different during heat stress. In addition, the venular diameter was not significantly different among the treatment groups (Fig. 3D).

Changes of arteriolar flow rate: The arteriolar flow rates in the vehicle and Xuebijing + HS groups were significantly lower than that in the control group when the core temperature increased to 38 °C during heat stress (*P* < 0.01). When the core temperature further increased to 41 °C, the arteriolar flow rates in the SOD + HS and Xuebijing + HS groups both decreased significantly compared with that in the control group (SOD + HS group: *P* < 0.05; Xuebijing + HS group: *P* < 0.01). The arteriolar flow rates in the Xuebijing + HS and SOD + HS groups were significantly higher than that in the vehicle + HS group when the core temperature was 38 °C. However, the arteriolar flow rate in all the rats exposed to heat stress was not significantly different among treatment groups when the core temperature increased to 41 °C (Fig. 3E).

Changes of venular flow rate: The venular flow rates in all the three groups exposed to heat stress were significantly lower than that in the control group when the core temperature increased to 38 °C during heat stress (vehicle + HS group: *P* < 0.05; SOD + HS and Xuebijing + HS groups: *P* < 0.01). When the core temperature further increased to 41 °C, the venular flow rates in the vehicle + HS and Xuebijing + HS groups both decreased significantly (*P* < 0.01), while the rate in the SOD + HS group did not decrease significantly. The venular flow rates in the Xuebijing + HS and SOD + HS groups were also significantly higher than that in the vehicle + HS group when the core temperature was 41 °C (Fig. 3F).

### Changes of average arterial pressure after heat stress

The average arterial pressure in the vehicle + HS and Xuebijing + HS groups decreased when the core temperature increased from 36 °C to 38 °C. However, the average arterial pressure increased spontaneously when the core temperature increased from 38 °C to 39.5 °C. When the core temperature further increased to 41 °C, which was considered to represent severe heat stroke, the average arterial pressure decreased again until the rats died; the blood pressure in the Xuebijing + HS group was significantly higher than that in the vehicle + HS group (*P* < 0.05).

**Figure 4:**
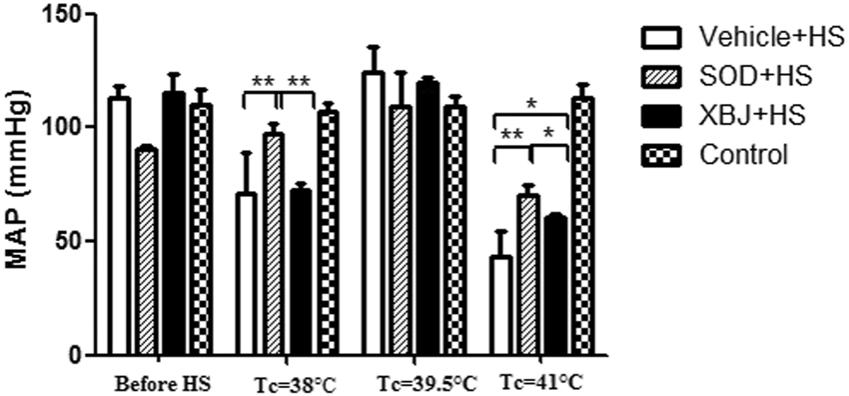
Mean arterial pressure (MAP). Right femoral artery catheter was performed before heat stress, and mean arterial pressure (MAP) was observed continuously. The MAP of rats in the three groups was compared at different time points. Rats in control group didn’t be subjected to continuous heat stress in an infant incubator, but were observed at same time of heat stress group rats. *P < 0.05; **P < 0.01, comparison between treatment groups. ^#^*P* < 0.05; ^##^*P* < 0.01, comparison between rats in the same treatment group at different time points.

By contrast, the average arterial pressure in the SOD + HS group was not significantly different from the levels in the other groups before heat stress. In addition, the average arterial pressure in this group also showed no evident decrease during heat stress. When the core temperature of rats increased from 36 °C to 38 °C (T2), the average arterial pressure in this group was significantly higher than that in the vehicle + HS and Xuebijing + HS groups (vehicle + HS group vs SOD + HS group: *P* = 0.000; SOD + HS group vs Xuebijing + HS group: *P* = 0.000). When the core temperature increased from 38 °C to 39.5 °C, the average arterial pressure among the three groups exposed to heat stress was not significantly different. When the core temperature further increased to 41 °C, the average arterial pressure in all three groups exposed to heat stress decreased sharply until the rats died. Interestingly, the blood pressure in the Xuebijing + HS and SOD + HS groups was still higher than that in the vehicle + HSgroup (Fig. 4).

### Loss of body mass in the four groups

**Figure 5:**
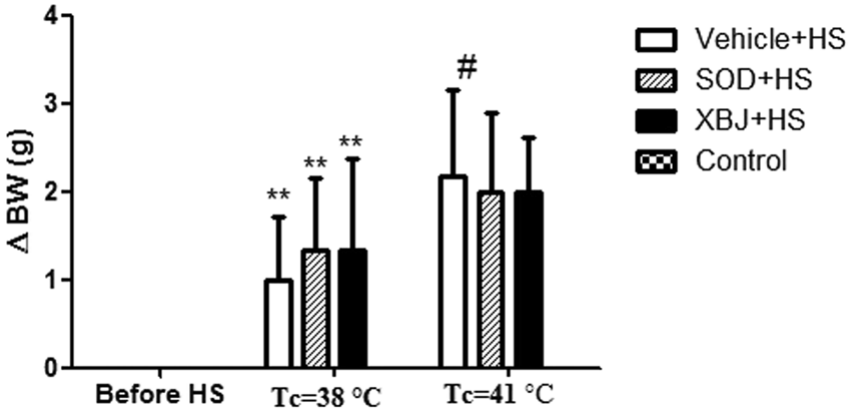
Changes of body mass. The loss of body weight (ΔWt) = Body mass before heat stress Body mass after heat stress. Rats in control group didn’t be subjected to continuous heat stress in an infant incubator, but were observed at same time of heat stress group rats. *P < 0.05; **P < 0.01, compared with the body weight before heat stress. ^#^*P* < 0.05; ^*##*^P < 0.01, compared with the body weight loss at Tc = 38 °C.

Significant body mass loss was evident in all three groups exposed to heat stress compared with the control group (*P* < 0.05). However, the body mass loss in the three groups exposed to heat stress, but not the control group, was not significantly different at the same core temperature (Fig. 5).

### Survival time

**Figure 6:**
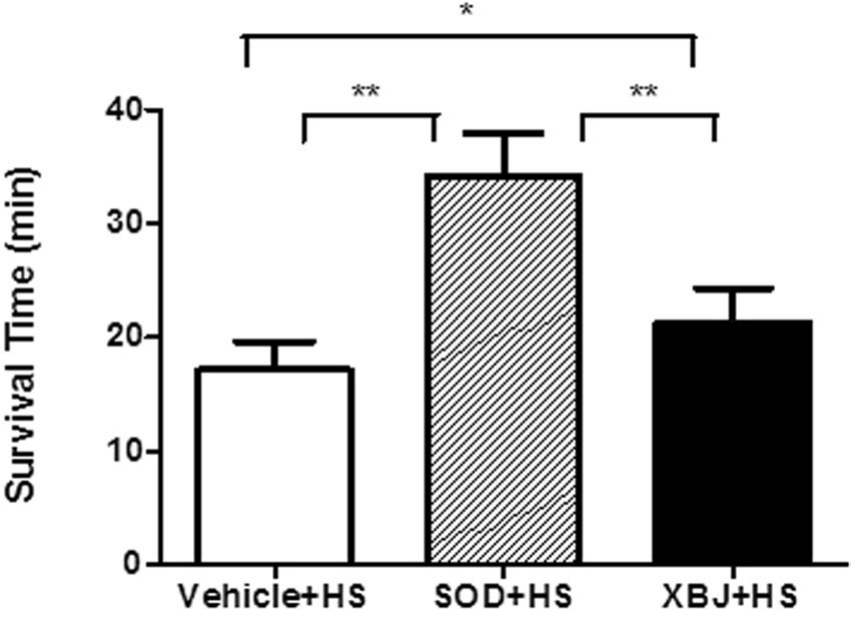
Survival time after Tc = 41 °C. The survival time after Tc = 41 °C was defined as the survival period from the time the core temperature of rats reached 41°C until death. *P < 0.05; **P < 0.01 compared between groups.

The survival time of rats in the SOD + HS group was significantly longer than that in the vehicle + HS and Xuebijing + HS groups when the core temperature increased to 41 °C (*P* < 0.01). In addition, the survival time in the Xuebijing + HS group was also significantly longer than that in the vehicle + HS group at this core temperature (*P* < 0.05) (Fig. 6).

## Discussion

Heat stroke can be classified as classic heat stroke or exertional heat stroke according to the etiology^4^. According to a previous study, severe heat shock was defined as follows in the present study: when persistently exposed to high temperature, high humidity environment, the core temperature of the animalsexceeded 42 °C and the MAP decreased by 25 mmHg from the peak level^19^. However, in this study, severe heat stroke occurred when the core temperature reached 41 °C. This is likely because the rats used in previous studies weighted 220–250 g. However, the spinotrapezius muscle and fascia were too thick in rats of this weight, and hence observing the microvessels with a microscope was extremely difficult. Therefore, rats weighed 160–180 g were used in this study after multiple preliminary studies. However, the relatively low body weight of these rats also created another disadvantage in that their resistance to heat stress was also relatively low.

The findings of this study showed that SOD activity decreased and the ROS level increased gradually in the spinotrapezius muscle of rats as the core temperature increased during heat stress. Recent studies have shown that SOD can relieve the inhibitory effects of superoxide on catecholamine, restore the reactivity of sympathetic vasoconstrictive agents, improve the symptoms of sepsis shock, maintain intracellular calcium homeostasis, improve mitochondrial functions, reduce oxygen radical damage, and restore homeostasis affected by shock^20,21^. Various animal models of shock have shown that the imbalanced oxidative stress response caused by decreased SOD activity is closely associated with multiple pathophysiological processes and microcirculation disturbance. Supplying SOD can effectively reduce organ function damage during shock^22^. However, the ROS generation catalyzed by various enzyme systems is closely associated with endothelial dysfunction. The ROS generated by these enzyme systems can cause oxidative stress, thus inducing mitochondrial dysfunction, endothelial dysfunction, vascular adhesion molecule abnormalities, increased permeability, inflammatory mediator release, and finally organ dysfunction^23^. In our study, the changes in the oxidative stress level were closely associated with local microcirculation disturbances, such as decreased microvascular blood flow and abnormal vascular reactivity. Supplying SOD to the rats effectively inhibited ROS generation during heat stress, thus improving the local microcirculation disturbances caused by oxidative stress.

The present study showed that Xuebijing could decrease ROS production by increasing glutathione and SOD levels in serum^24^. The findings of this study also demonstrated that the SOD activity increased gradually in the spinotrapezius of rats injected with Xuebijing, and Xuebijing injection also effectively decreased the ROS level in the local spinotrapezius tissues.

In this study, at the onset of heat stress, the MAP decreased significantly; this is likely because body fluid loss and the regulation of body fluid distribution in the early stage of heat stress can lead to a decrease in the effective circulating blood volume. The hemodynamic characteristics of heat shock are similar to those of hemorrhagic shock.

The findings of this study showed that in the early phase of heat stress (Tc = 38 °C), the arteriolar and venular V_RBC_ and blood flow rate decreased significantly, which was in agreement with the findings in the early stage of hemorrhagic shock. Previous studies have shown that in the early phase of hemorrhagic shock, the microvessel V_RBC_ and blood flow rate decrease^18^. In our study, when the rats were pretreated with Xuebijing or SOD, the V_RBC_ and blood flow rate in microvessels were significantly better than those in the vehicle group, suggesting that Xuebijing could protect against the heat-stress-induced decrease in the blood flow rate in microvessels.

In this study, however, the changes in the arteriolar and venular diameters from the initiation of heat stress to the onset of severe heat stroke were not statistically significant. Previous studies on hemorrhagic stroke showed that the arteriolar and venular diameters decreased (indicating microvessel contraction) in hemorrhagic stroke^18^. It is known that during shock, the vasoconstriction of vessels of the skin favors the shifting of blood toward more crucial organs such as the heart, brain or liver. Nevertheless, a recently study demonstrated that maintenance of functional capillary density was the only critical microvascular parameter correlated with survival in severe hemorrhagic shock^26^. In our study, we also found that after heat stroke (Tc > 41 °C), systemic hemodynamic changes were closely related to the decrease in the microcirculatory blood flow rate. Thus, our findings further suggested that the decrease in the blood flow volume was mainly caused by the decrease in blood flow velocity in the microvessels in the period from the initiation of heat stress to the onset of severe heat stroke.

This study found that the MAP decreased in the early stage of heat stress. We speculated that the MAP decrease in this phase could be associated with self-regulation, such as the re-distribution of body fluid. However, the MAP was restored to the level observed before heat stress when the core temperature increased to 39.5 °C. This restoration could be associated with the occurrence of heat stress responses. The MAP was maintained at a normal level until the core temperature reached 41 °C, at which time severe heat stroke occurred, and the rats subsequently died. Previous studies on stroke^12^ showed that heat stress could promote cellular metabolism and reduce visceral blood flow, thus inducing ischemia and hypoxia in the intestine and liver cells. Therefore, visceral hypoperfusion, as well as changes in immune functions and intestinal barriers, could occur in heat stress. In addition, endotoxin leakage and inflammatory factor release also increased, and vascular endothelial growth factors (such as NO and endothelin) were released. The cytokines and inflammatory factors induced by high temperature could influence sweating and vascular bed changes to affect thermal regulation mechanisms and alter vascular permeability and visceral microcirculation, thus causing hypotension, high fever, and even multiple organ dysfunction. Out study further suggested that after heat stroke (Tc > 41 °C), microcirculatory and systemic hemodynamics changed significantly, and the blood flow rate and MAP decreased at the same time point, but the MAP was significantly higher in rats pretreated with Xuebijing and anti-oxidants than in rats in the vehicle group when the core temperature was more than 41 °C, suggesting that Xuebijing had protective effects on the systemic hemodynamics of rats, although the effects were not as pronounced as those of anti-oxidants. These findings indicated that the clinical application of anti-oxidants or Xuebijing could be an effective method for treating severe heat shock.

This study also showed that the microcirculatory blood flow volume (including the arteriolar and venular volumes) and the MAP decreased in the early stage of heat stress. However, when the core temperature reached 38–39.5 °C, the MAP was restored to the level observed before heat stress. By contrast, the microcirculatory blood flow volume did not recover with the improvement in systemic hemodynamics; instead, the blood flow volume decreased progressively until the rats died. These findings suggested that in the heat stress model, microcirculation disturbances appeared before systemic circulation disturbances, and the persistence of microcirculation disturbances could be an important factor underlying the difficulty in treating shock and its poor prognosis.

Early studies have shown that dehydration is also a typical manifestation of stroke^4^. This study showed that with the increase in temperature, the rats in the three groups all showed mild-to-moderate body fluid loss. The volume of body fluid loss at the core temperature of 41 °C was approximately 1–3% of the total body weight. However, providing normal saline or SOD before heat stress induction did not improve the dehydration. We speculate that the mild-to-moderate dehydration was not associated with the prognosis of severe heat shock in rats.

The findings of this study showed that SOD activity in local tissues decreased gradually during heat stress, while the level of ROS increased gradually, which was associated with poor prognosis. Aprevious study on sepsis^25^ showed that normal capillaries maintained homogenous blood flow from endothelial cells to precapillary arterioles; however, such blood flow could be blocked by sepsis. In addition, sepsis also disturbed the activation of endothelial cells, lymphocytes, and platelets. When the blood flow showed heterogeneous changes, a severe oxygen supply/consumption imbalance appeared. In other words, microcirculation disturbances could be an important factor causing multiple organ dysfunction and elevated mortality. Thus, our findings are similar to those of previous studies. We also found that using Xuebijing or anti-oxidants effectively inhibited the increase in ROS during heat stress and improved the microcirculation, thus improving the survival time of rats after severe heat shock and finally improving the prognosis of rats with severe heat shock.

In summary, in the present study, we continuously observed the microcirculation in the spinotrapezius of rats with severe heat shock and measured the total SOD activity and ROS level changes. The findings revealed the changes in the oxidative stress levels in local tissues during heat stress, showed an association between the changes in the microcirculatory blood flow volume and those in the systemic circulatory blood flow volume, and demonstrated that a gradual decrease in SOD in local rat tissues could result in increased ROS levels, which in turn induced microcirculation disturbances. The microcirculation disturbances occurred before systemic circulation disturbances and could be an important factor causing circulatory failure after severe heat shock. Xuebijing reduced the local ROS level and protected the microcirculation during heat stress, thus improving the prognosis of rats with severe heat shock.

## Materials and Methods

### Animals

Sixty-seven male Wistar rats of specific-pathogen-free grade and weighing 160–180 g were used in this study. Standard food and water were provided to the rats during the study. All the rats were fasted from food but not water for 12 h before the experiments. Forty of the rats were divided into 4 groups (10 in each group) as follows: control, Vehicle + HS, SOD (cat number: S5395SOD, Sigma, USA) pretreatment + HS (SOD + HS), and Xuebijing pretreatment + HS (Xuebijing + HS). The grouping of rats is shown in Table 1. The rats in the SOD + HS group were injected with 5 mg/kg SOD (SOD was first diluted to 4 mL with normal saline)^15^, while the rats in the Xuebijing + HS group were injected with 4 ml of Xuebijing^14^. All the reagents were injected via the tail vein and allowed to circulate for 1 h before the subsequent experiments. All the rats except for those in the control group were placed in an infant incubator for heat stress exposure. The temperature and humidity in the infant incubator were set at 40 °C and 65%, respectively^16^. The anal temperature of rats was measured every 10 min after the initiation of heat stress. The rat body weights were also measured before heat stress, at the core temperature of 38 °C, and at the core temperature of 41 °C. The survival time of rats was assessed when the core temperature reached 41 °C. Twenty-seven rats were selected for SOD activity and ROS level measurements. The 27 rats were divided into 3 groups as follows: HS, SOD, and Xuebijing. In each group, the rats were further divided into three subgroups as follows: control, core temperature 38 °C, and core temperature 41 °C. Three rats from each group were sacrificed when the core temperature reached 38 °C and 40 °C, along with three rats from the control group. The spinotrapezius muscle was obtained for SOD activity and ROS level measurements. The grouping of rats for SOD and ROS measurements is shown in Table 2.

**Table 1:** Grouping and number of animals for microcirculatory observation.

	Control	Vehicle + HS	SOD + HS	Xuebijing + HS	Total
Microcirculation observation	10	10	10	10	40
Total	10	10	10	10	40

**Table 2:** Grouping and number of animals for SOD and ROS detection.

		Vehicle	SOD	Xuebijing	Total
Detection of SOD and ROS	Control	3	3	3	9
	Tc = 38 °C	3	3	3	9
	Tc = 41 °C	3	3	3	9
Total		9	9	9	27

This study was conducted in the Guangdong Key Laboratory for Shock and Microcirculation (at Southern Medical University). All the animals were deeply anesthetized by pentobarbital sodium (60 mg/kg) injection, the routine animal anesthesia method in this study, before the operation. This study was approved by the Animal Ethics Committee of Southern Medical University and conducted according to the guidelines and regulations for the use and care of experimental animals in China. In addition, this study also minimized the number of animals and the discomfort of the animals, according to the guidelines for experiments with living animals in China.

### Measuring the total SOD activity in the spinotrapezius

Spinotrapezius tissues were obtained, and nine volumes of normal saline was added (9 ml of normal saline was added to 1 g of tissue). Then, the tissues were cut into small pieces, homogenized in an ice-water bath, and centrifuged at 2500–3000 rpm for 10 min. The supernatant of the 10% homogenate was collected, and the protein concentration was quantified using a bicinchoninic acid (BCA) kit. The SOD inhibitory rate was calculated using the following equation:

$$\begin{array}{c}{\rm{SOD}}\,{\rm{inhibitory}}\,{\rm{rate}}\,( \% )=[({\rm{Acontrol}}-{\rm{Acontrol}}\,{\rm{blank}})-({\rm{Asample}}-{\rm{Asample}}\,{\rm{blank}})]\\ \quad \quad \,/(\mathrm{Acontrol}-\mathrm{Acontrol}\,{\rm{blank}})\times \mathrm{100} \% \end{array}$$

SOD activity was calculated using the following equation:

$$\begin{array}{c}{\rm{SOD}}\,{\rm{activity}}\,(U/\mathrm{mg\; prot})\\ \quad =\,{\rm{SOD}}\,{\rm{inhibitory}}\,{\rm{rate}}\div{\rm{dilution}}\,{\rm{ratio}}\,{\rm{of}}\,{\rm{the}}\,\mathrm{50} \% \,\mathrm{reaction}\,\mathrm{system}\,(0{\rm{.24}}\,\mathrm{mL}/0{\rm{.02}}\,\mathrm{ml})\\ \quad \,\,\div{\rm{protein}}\,{\rm{concentration}}\,{\rm{of}}\,{\rm{the}}\,{\rm{sample}}\,({\rm{mg}}\,\mathrm{prot}/\mathrm{mL}){\rm{.}}\end{array}$$

### Measuring the ROS level in the spinotrapezius

The weight of the spinotrapezius muscle was accurately measured. Homogenization medium (100 mmol/L phosphate-buffered solution) was then added at a ratio of 1:20 [weight (g):volume (mL)], and the muscle was homogenized manually in an ice-water bath to obtain single-cell suspension. The cell density was adjusted to 10^6^ cells/mL. The suspension was centrifuged at 1000 g for 10 min, and the supernatant was collected. Part of the supernatant was used to determine the protein concentration by the BCA method. Then, 1 mmol/L fluorescein DCFH was added to each sample and incubated at 37 °C for 30 min. A flow cytometer was used to analyze ROS levels. The excitation wavelength was 485 nm, and the emission wavelength was 525 nm (FITC).

### Microcirculation monitoring

Preparation of the spinotrapezius muscle and intravital microscopy: The spinotrapezius muscles are located anatomically in the mid-dorsal region, originating in the lower thoracic and upper lumbar regions and inserted at the scapular spine. Spinotrapezius muscles were prepared as previously described by Gray^17^. Briefly, the spinotrapezius muscle was exteriorized with marginal damage to the fascia. No evidence of local trauma, which might impact regional blood flow in this model, was reported. Throughout the surgical procedure and course of the experiment, the exposed tissue was continuously perfused with a heated Krebs–Henseleit bicarbonate-buffered solution to maintain a constant temperature of 37 °C, as well as the humidity, pH, and ionic strength of the sample. The solution was heated to prevent loss of heat via perfusion. The exposed spinotrapezius muscle was fixed at six equidistant sites around the caudal periphery to ensure consistent shape and length of the selected vessels. Unbranched, third-order arterioles and venules (diameter range 20–60 μm) were selected at random and videotaped at 2000 frames/s using a CR5000X2 high-speed camera (Optronis, Germany). The vessel diameters and red blood cell velocities (VRBC) were assessed at a rate of 25 frames/s^18^. Assuming cylindrical geometry, the blood flow rate was calculated using the following formula: π × Vmean × (D/2)2; Vmean = VRBC/1.6 and D = diameter of the vessel^18^.

**Figure 7:**
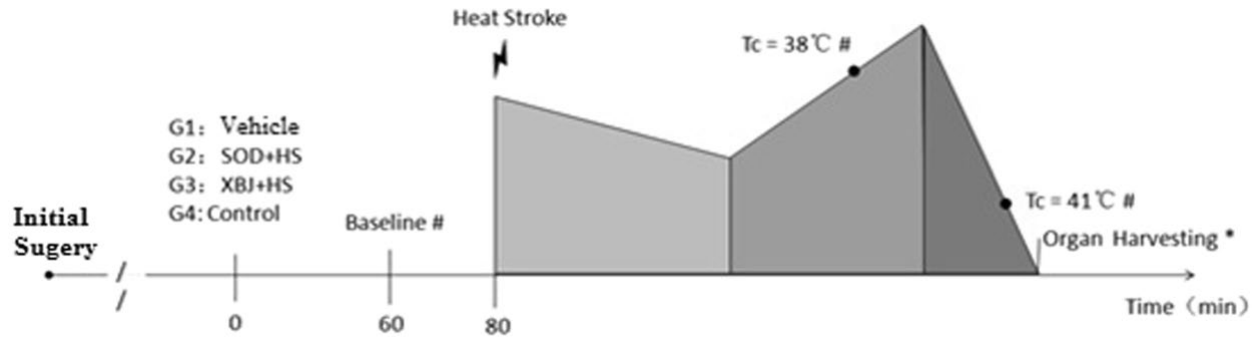
Experimental protocol and outline. Surgery included spinotrapezius preparation and right femoral artery catheterization. Mean arterial pressure was detected for 20 min continuously until it was stable. The microcirculation was observed by intravital microscopy. The arteriolar/venular V_RBC_ and diameter were detected at this time point. All rats were kept in an infant incubator for heat stress (environmental temperature: 40 °C, humidity: 65%).

The prepared spinotrapezius muscle was covered with thin gauze, which was continuously perfused with a heated Krebs–Henseleit bicarbonate-buffered solution. Then, right femoral artery catheterization was performed. The mean arterial pressure (MAP) was observed continuously for at least 20 min until it was stable. SOD and Xuebijing were injected accordingly as described earlier. One hour later, the rats were placed in an infant incubator for heat stress exposure, and the microcirculation was observed by intra vital microscopy. The arteriolar/venular VRBC and vascular diameter were detected in the control group before heat stress and when the rectal temperature reached 38 °C (Tc = 38 °C) and 41 °C (Tc = 41 °C, representing the group with severe heat stroke). The survival time and the changes in rat body weight were calculated after Tc = 41 °C. Since the rats had to be removed from the infant incubator at each time point, the microcirculation observation was conducted as fast as possible to avoid allowing the core temperature of the rats to drop (Fig. 7).

### Weight change and survival time after exposure to Tc = 41 °C

The body weight of rats was measured before heat stress and at Tc = 38 °C and 41 °C. The change of weight was calculated as follows: (weight before heat stress – weight when Tc = 38 °C/41 °C)/weight before heat stress × 100%. The survival time analysis was started immediately when the rectal temperature reached 41 °C.

### Statistical analysis

All data were expressed as the means ± standard deviation (SD). Analysis of variance for repeated measurements was used to compare the differences between groups at different time points. Pair wise comparisons were conducted using t tests. The level of statistical significance was set at *P* < 0.05. All data were analyzed using IBM SPSS software (IBM SPSS 19, IL, USA).
